# Modern Electrospray Ionization Mass Spectrometry Techniques for the Characterization of Supramolecules and Coordination Compounds

**DOI:** 10.1021/acs.analchem.4c01028

**Published:** 2024-04-30

**Authors:** Niklas Geue

**Affiliations:** Michael Barber Centre for Collaborative Mass Spectrometry, Manchester Institute of Biotechnology, Department of Chemistry, The University of Manchester, 131 Princess Street, Manchester M1 7DN, United Kingdom

## Introduction

Mass spectrometry (MS) has emerged as an integral part of the analytical toolbox and is one of the most routinely used techniques. It has been historically well explored for small organic compounds, as such molecules only contain covalent bonds and are mostly easy to ionize.^1^ In the past decades, the focus of MS has expanded rapidly, and two areas have shown particularly big promise: the analysis of complex mixtures, e.g., in “omics”,^2,3^ and the structural characterization of biomacromolecules in their native state.^4,5^

Between the extremes of small (organic) molecules and biomacromolecules, the analysis of synthetic inorganic molecules is of particular interest to chemists, including noncovalently bound supramolecules and labile coordination compounds. These systems have become increasingly important in application areas such as medicine, catalysis, and materials as well as in mimicking the functionality of biomolecules.^6^ The structural characterization of such assemblies is challenging, as X-ray crystallography, NMR spectroscopy, or computational methods are often difficult or not feasible for larger structures.^6^

Methods adapted from native MS offer a range of advantages for their characterization over other methods: (a) an ultrahigh mass resolution that enables the unambiguous identification of composition and stoichiometry;^5^ (b) the robust analysis of complex product mixtures, where different compounds are separated by their *m*/*z*; (c) the hyphenation with other techniques such as chromatography,^7,8^ ion mobility,^9,10^ or spectroscopy;^11,12^ and (d) the potential to adapt characterization workflows to an industrial scale if appropriate.^6^ The characterization of supramolecular and coordination compounds using MS has been reviewed regularly,^6,13−25^ including a book published by Schalley and Springer in 2009;^26^ however, MS is still not commonly and always confidently used in synthetic inorganic laboratories.

This tutorial will focus on the practical application of MS (mostly electrospray ionization, ESI) for such inorganic complexes, beginning with technical details as well as potential challenges. The analysis of MS data will further be discussed in detail, before the tutorial ends with an introduction to other gas phase techniques suitable for the investigation of such compounds. While the characterization of these inorganic systems with MS is less trivial than for small organic compounds, every mass spectrometrist, chemist, and technician can learn the necessary skills with the help of this tutorial.

## Technical Details

**Figure 1:**
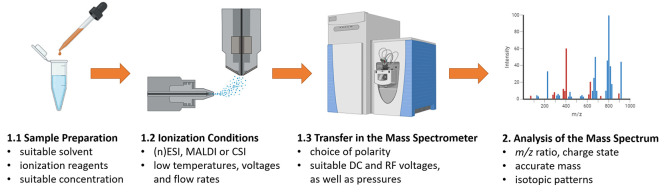
Mass spectrometric analysis of supramolecules and coordination compounds, including sample preparation, ionization conditions, ion transfer, and analysis. Abbreviations are as follows: (n)ESI, (nano)-electrospray ionization; MALDI, matrix-assisted laser desorption ionization; CSI, coldspray ionization; DC, direct current; RF, radio frequency; *m*/*z*, mass to charge ratio. Designed with BioRender.

The main aim of the mass spectrometric analysis is to obtain a mass spectrum of the synthetic product. This section will be focused on technical details to facilitate this process and on pitfalls to avoid (Figure 1).

### Sample Preparation

Synthetic products are usually provided as solids or as reaction mixtures in solution, and the most common ionization methods start from solution. For solid samples, the choice of solvent is crucial. As it is challenging for a mass spectrometrist or technician to understand the intrinsic properties of the product on the same level as the synthetic chemist, ideas for solvent choices are a valuable input that should be provided. Suitable solvents entirely depend on the reaction product and its properties—the sample (and potentially the ionization reagent) needs to be dissolved easily while maintaining suitable concentrations, but without inducing unwanted disassembly reactions. For labile compounds, it is often advisable to prepare the solution fresh prior to analysis, which minimizes the time for disassembly or other reactions. It is also important to choose a solvent that is compatible with the ionization method of choice. For ESI, this usually involves polar solvents such as water, methanol, and acetonitrile, whereas nonpolar solvents are less suitable.^27^

For ESI, it is often useful to add salts such as alkali metal halides, or acids/bases, depending on the product and its lability. This approach can help to enhance the signal of adduct ions [M + *x*A]^*x*+^ and [M + *y*A]^*y*−^ (with A^+^/A^–^ being, e.g., an alkali metal cation or halide anion), or of the protonated and deprotonated species [M + *x*H]^*x*+^ and [M – *y*H]^*y*−^, respectively. Buffer solutions to control the pH value, such as ammonium acetate that is often used in biological mass spectrometry, are usually not needed.^28^

The analyte concentration in solution is important, and this is particularly difficult to control when a reaction mixture is the starting point rather than a solid. Modern mass spectrometers are highly sensitive, and the optimal sample concentration is decreasing constantly.^29^ Typical analyte concentrations in modern MS lie in the low micromolar region.

*What are the consequences of using too high concentrations?* One problem is that higher concentrations can cause crystallization in the solution and at the air–water interface, and when using ESI and nanoelectrospray ionization (nESI), this can often clog the capillary.^30,31^ High concentrations also lead to clustering during the ESI process, and this makes the spectrum more complex and may also not reflect the molecules present in solution.^19^ Another potential problem is space-charging effects, which are due to the repulsion of charged ions with the same polarity. This pheomenon can lead to a decrease in accuracy, sensitivity, and resolution of the mass^32,33^ (and ion mobility^34,35^) measurements, although in practice this is usually not a problem for mass. High concentrations lead more likely to oversaturation of the detector,^36^ which decreases their lifetime and also means that instruments have to be cleaned more frequently due to the contaminations with neutrals from solution.^23^

*What are the consequences of using too low concentrations?* Below a certain threshold, the sensitivity of mass spectrometers is not sufficient to detect the analyte. Less obvious is that the formation of coordination compounds and supramolecules is often directed by self-assembly, and this is driven by entropic factors that in turn depends on concentration.^23,37,38^ Hence, synthetic products can disassemble in solution because the concentration is too low. Most MS efforts are pointless if the analyte is not present in solution, and for some compound classes, the consequences of using high concentrations have to be accepted in order to maintain the analyte in solution. For metallosupramolecular complexes, typical solution concentrations to avoid disassembly are in the high micromolar and low millimolar region.^37,38^

It is advised to start at the concentration that is considered ideal for the sensitivity of the instrument. In case the compound relies on self-assembly, and if only smaller fragment peaks are observed but not the analyte signal, the user should gradually increase the concentration. This should lead to the occurrence of larger fragments and eventually the analyte; however aggregates can form at too high concentrations that exceed the mass of the analyte. If no analyte ions are found, and if no changes occur while increasing the concentration further, the analyte absence cannot be explained with solution disassembly.

### Ionization

The use of soft ionization methods has enabled the characterization of large biomacromolecules,^39^ and the lessons learned from biological MS can be applied to labile inorganic substances.^6^ The most commonly used ionization source is ESI, where a high voltage is applied to a solution in a capillary. Ions in the liquid migrate to the surface until coulumbic repulsion overcomes the surface tension and an ion–solvent cone forms at the tip of the capillary. The detailed mechanism of ionization depends on the size and structure of the molecule and remains debated;^40−42^ however both the ionization energy/electron affinity as well as the surface activity play an important role in determining how many ions are formed per analyte species in solution (“response factor”).^43^

A further adaptation of ESI for the ionization of labile complexes to the gas phase is nano-ESI,^44^ for which glass capillaries with a sharp opening in the (sub)micron regime are used.^45^ Due to lower flow rates and voltages, and the possibility of lower source temperatures, very large structures such as whole viruses can be ionized, and hence no relevant, upper mass limit exists for synthetic chemists with the exception of large polymers.^46^ The design of these “nano-tips” needs some consideration, and depending on usage, either the purchase of premade nanotips or the in-house design with capillary pullers is possible. Parameters for common nano-ESI tips can be found elsewhere.^45,47^

For successful ionization using nESI, a voltage needs to be applied to the solution inside the tip. Two common options exist: the insertion of an inert metal wire (often Pt) that is connected to the source, or the coating of the nanotip with a conductive material (often Au, Pd or Pt).^48,49^ For most cases, both methods are equivalent. The disadvantage of nESI compared to ESI are the difficulty to hyphenate the technique with liquid chromatography, although this has been partially overcome,^50^ and a tedious tip preparation. Nanotips are also not perfectly reproducible, which can make the development of robust workflows difficult.^45^

Source conditions for labile inorganic complexes need to be soft, which means to avoid in-source fragmentation and to preserve the structure of the analyte. Low flow rates, temperatures, and voltages are recommended.^23,51^ There is a trade-off between these soft parameters and those that maximize signal (high flow rates, temperatures and voltages), and finding the ideal parameters may require careful tuning. A key step in the formation of ions via ESI is desolvation, which benefits from high pressures and temperatures in the ion source, and also depends on the solvent.^52^ Incomplete desolvation can lead to solvent adducts, which particularly occur with coordinating solvents such as acetonitrile.^19^ The source temperature is a major factor for preserving the original structure of labile molecules during ESI. Cryo- or cold-spray ionization sources (CSI) have been developed, achieving good results that seem not easily amenable with ESI.^53−56^ As CSI is not commercially available and only few sources exist, it will not be discussed further.^57,58^

Another ionization technique is matrix-assisted laser desorption ionization (MALDI), which is often deployed for synthetic molecules such as organic polymers^59^ and dendrimers.^60^ An important difference to ESI is that fewer multicharged ions are formed, which facilitates data interpretation. The sample is embedded in an organic matrix and excited with short laser pulses. After the energy absorption through the matrix and relaxation in the crystal lattice, parts of the sample are desorbed and ionized, and transferred to the mass spectrometer.^61^ While MALDI has its merit for labile synthetic molecules, it is far less common than ESI. Other ionization methods, such as electron ionization, chemical ionization, or field ionization, are even less frequently used but can be suitable, e.g., for organometallic compounds.^62^

### Ion Transfer in the Mass Spectrometer

The transfer of ions in the mass spectrometer is realized through a combination of direct current (DC) and alternating radiofrequency (RF) fields.^63^ The most important consideration is the polarity of the ion optics, as this determines whether cations or anions are transmitted. The preference for either polarity depends on the nature of the sample, its acidity/basicity, and its ionization energy/electron affinity.^64^ Biological MS relies overwhelmingly on the positive ion mode; however, for synthetic molecules, negative ion mode is also frequently used. MS data collected in negative ion mode are often simpler, less intense, and less noisy, due to the availability of fewer ionization pathways.^19^

There is a trade-off between high ion transmission (using high voltages and low pressures) and preserving the ion structure (using low voltages and high pressures), and the required “softness” of the parameters depends on the rigidity and stability of the sample. The *m*/*z* also plays an important role in how well ions are transmitted. Higher *m*/*z* ions require higher RF voltages (and lower RF frequencies, but this is usually not user-controlled), but this is not specific for inorganic compounds. More details on tuning mass spectrometers can be found in resources for the specific instruments.^63^ As modern mass spectrometers are able to transmit ions in the megadalton regime (1 Dalton = 1 Da = 1 g·mol^–1^), the size of synthetic compounds should rarely be a limiting factor for ion transfer when properly tuned.^46^

## Analysis of the Mass Spectrum

The measurement of the mass to charge ratio (*m*/*z*) is realized in the mass analyzer. The most common mass analyzers utilized in modern, commercial mass spectrometers are quadrupoles, time-of-flight (TOF) analyzers, and ion traps.^1^ Quadrupoles have the lowest resolution of the ones listed above and are mainly used for *m*/*z* selection in tandem mass spectrometry experiments or when only a narrow *m*/*z* window is sufficient for analysis. Both TOF and ion traps can have significantly higher resolution than quadrupoles. A well-known example of ion traps is the orbitrap, which delivers high resolution with moderate costs and effort and has hence emerged as a game changer in modern mass spectrometry.^65^ More detailed information about mass analyzers can be found elsewhere.^1^

### *m*/*z* and Mass

**Figure 2:**
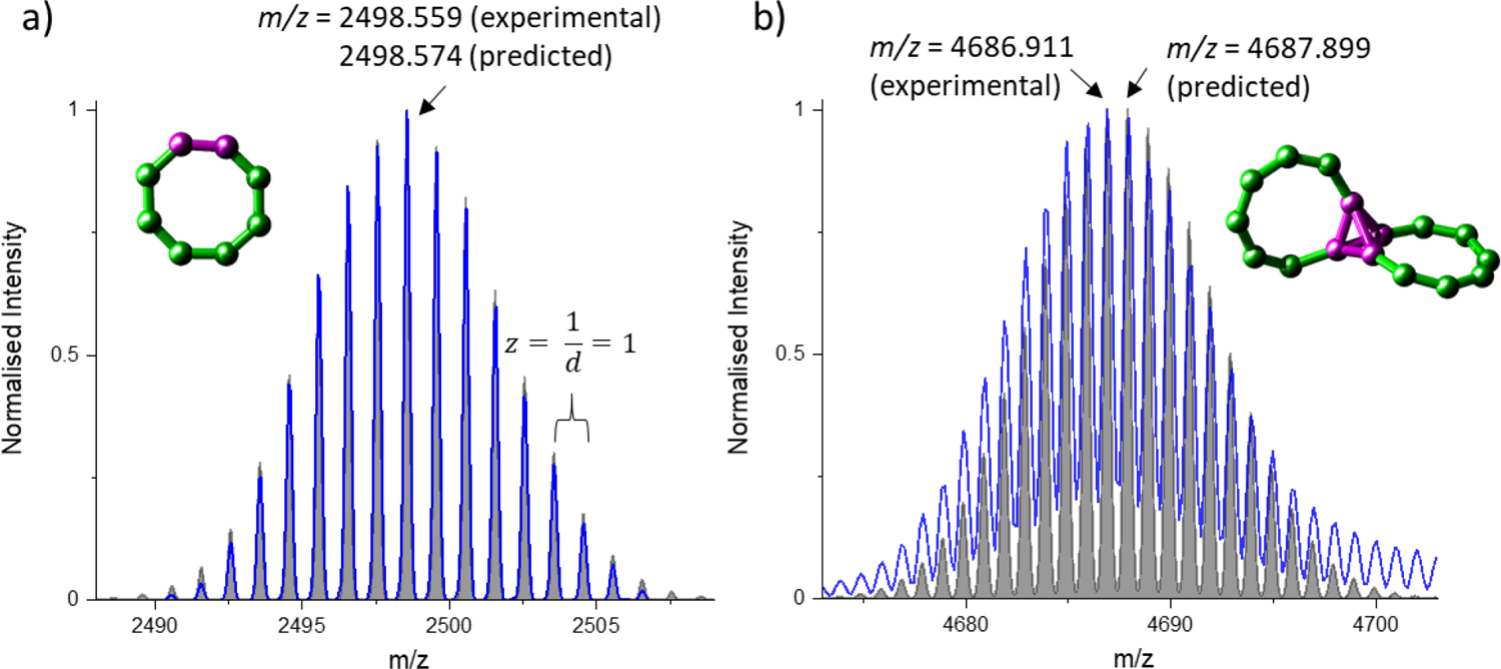
Experimental (blue) and predicted (gray) isotopic patterns of (a) [Cr_6_Gd_2_F_8_(O_2_C^*t*^Bu)_16_NH_2_^*n*^Pr_2_]^+^ and (b) [Cr_12_Gd_4_F_21_(O_2_C^*t*^Bu)_28_(NH_2_^*n*^Pr_2_)_2_]^+^. Example a) illustrates how the charge state can be determined by quantifying the *m*/*z* distance between two neighboring isotopic peaks, leading to *d* = *z* = 1. The predicted isotopic pattern agrees well with the experiment, and so does the accurate mass of the most intense peak. Case b) is more difficult, and the unexperienced reader might consider the agreement between the experiment and prediction sufficient. While the digits of the accurate mass are in good agreement with simulation, the experimental maximum and the whole distribution is shifted to lower *m*/*z*. The agreement is not sufficient to confidently assign this peak to the proposed sum formula. Based on X-ray crystallography data, we found that some of the fluoride atoms have likely been substituted for hydroxyl groups, which suggests an overlap of ions with different numbers of F atoms and OH groups present.^37^ Predicted isotopic patterns were simulated with enviPat^67^ based on a ThermoFisher QExactive UHMR at a resolution of 12 500 (as experiment). Adapted with permission from ref (37). Copyright 2023, The Authors.

High-resolution mass spectra provide wide and rich information that goes far beyond the comparison of measured mass with predicted molecular weight. For multiply charged ions, the first step is decoupling the mass *m* from the charge *z*. High-resolution mass spectra of most small ions show isotopic distributions, and the distance *d* between two neighboring isotopic peaks is related to the charge state of the ion via the formula *d* = 1/*z* (Figure 2a for the example of a {Cr_6_Gd_2_} ring). Based on the charge state of the ion, the mass can easily be determined by multiplying *z* with the measured *m*/*z*. This approach usually works well, except for when a limitation in resolution or overlapping peaks occur. For the former, the resolution in the mass spectrometer may be increased, e.g., by extending the time the ions spend in the ion trap.^65^ Overlapping peaks can be addressed by deconvoluting the different signals, either manually or with software,^66^ or by using orthogonal separation approaches. A classic example of overlapping species is the combination of the monomer [M + *x*A]^*x*+^ and its dimer [2 (M + *x*A)]^2*x*+^, which share the exact same *m*/*z*. The only difference between both ion populations is the distance between the isotopic peaks; however, the pattern may not be easy to interpret by eye. Here, a simple deconvolution of the differently charged species is sufficient to distinguish both ions. More complicated is the separation of isomeric ions of the same charge state, and ion mobility can be useful for that purpose as it separates ions based on their size and shape.

### Resolution and Accurate Mass

Peak overlaps also occur between ions with similar, but not identical *m*/*z*, and for that the resolution of the instrument as well as the accurate mass play an important role. The former defines how well two given peaks can be separated, and continuous advances in commercial instrumentation have led to increasing resolving powers.^68,69^

The accurate mass is the experimentally determined mass of an ion with known charge, and the precision of this value depends on the accuracy and precision of the measurement.^70^ For modern orbitrap and TOF mass spectrometers, these ideally agree within 5 ppm (“parts per million”) compared to the predicted mass. Practically, this corresponds to a difference of one to three digits, depending on the size of the ion, the quality of the calibration, and the resolution of the instrument (Figure 2a for the example of a {Cr_6_Gd_2_} ring).

It might not be possible for synthetic chemists to control the calibration frequency or quality of an instrument; however, the mass accuracy can be determined by introducing a sample of known accurate mass (e.g., NaI or CsI clusters) as an internal standard, which can either give a rough indication of the current mass accuracy or can even serve as the basis for secondary calibration.^19^ It can also help to collect a mass spectrum just around the peak of interest, allowing to average as many ions as possible for better accurate mass measurements. The accurate mass can also predict the rough elemental composition of an ion based on the difference to the next full integer (“mass defect”).^70^ For molecules that involve only common organic elements such as H, O, N, and C, the accurate mass is usually close to the integer (±0.2 Da), whereas transition metals often have larger mass defects, and their ions are found in the whole range between two integer *m*/*z* units.

### Isotopic Distribution

High-resolution mass analyzers yield isotopic patterns for small molecules, which occur as most elements involve more than one abundant isotope. When many different atoms are combined, their combination yields a fingerprint-like pattern.^19^ Based on the ion’s sum formula and the natural isotopic abundances for a given element, this pattern can be simulated. Comparison of the experimental and simulated isotopic distribution can guide and confirm the peak assignment to a given formula. For small ions including elements with prominent isotopic distributions (e.g., Cl, S, Fe), the pattern can also reveal how many atoms of an element are present. Several online tools are available to predict isotopic distributions, and software from mass spectrometry vendors also often offers this possibility.^71,72^ It is essential to understand that averaged mass spectra, when acquired for a few minutes, can include up to hundreds of millions of ions. With such high ion counts, the statistical foundation of isotopic assignments is very powerful, and an isotopically resolved peak should match the simulated pattern almost perfectly (Figure 2a for the example of a {Cr_6_Gd_2_} ring). The isotopic pattern also depends on the natural isotope composition of the elements, ion–ion interactions, electronic noise, as well as the type of mass analyzer and its resolution.^73^ The last two are taken into account by some online resources and need to be considered in particular when using high resolution mass analyzers like the orbitrap.^67,74,75^

A problem occurs when peaks overlap, which results in a deviation from the expected isotopic distribution (assuming they have similar accurate masses of the isotopic peaks, otherwise they lie between the other ions’ signal), as shown in Figure 2b for a {Cr_12_Gd_4_} species. While this can be simulated,^76^ it is also sometimes possible to conclude by eye whether a given peak consists of one or more ions, based on peak shape (e.g., whether there is a “valley” in the isotopic distribution), and differences in the accurate mass.

Besides the advantages for peak assignment, the possibility to isotopically resolve peaks also opens a venue for distinguishing a compound from its isotopically labeled species (e.g., with ^2^H instead of ^1^H). This was for example used by Sawada et al. to analyze the preference of different host–guest complex enantiomers.^77^

### Analysis Approach

Most users assign the mass spectrum manually, although for frequent users software exists that facilitates this process significantly.^76,78^ For any assignment, the *m*/*z*, accurate mass, and isotopic distribution need to agree, and the formula needs to be a reasonable guess containing sensible components, oxidation states, coordination numbers, and the correct overall charge. The following procedure is recommended for the analysis of a mass spectrum of the molecule M:

1. Search for molecular adduct ions^79^ of the formula [M + *x*A]^*x*+^ (positive ion mode) or [M + *y*A]^*y*−^ (negative ion mode) with one or more charge carrying ions (A = H^+^, Na^+^, K^+^, Cl^–^, etc.). For negative ion mode, anions of the type [M – *y*H]^*y*−^ are also often found due to the loss of protons from acidic groups.

2. Look out for other charge states of the same intact analyte, that could originate from different numbers of charge carriers A^+^/A^–^ or through successive loss of oppositely charged counterions.

3. Are there other repeating patterns, separated by the same *m*/*z* ratio? These can indicate cluster formation or polymeric contaminations. Determine the *m*/*z* difference between two neighboring peaks, and try to assign the pattern. Although these assignments can inform on potential contaminations, it is often more important to avoid them rather than identifying them.

4. If you are unsure if a given peak originates from the analyte or is a contamination in the solvent, run a mass spectrum of the blank. Several contaminant ions are known for ESI-MS, however, often contaminations are sample-specific.^19^

5. Search for reasonable fragments, e.g., for complexes with loosely bound ligands, as well as ions with varying oxidation states of transition metals. These can occur due to reactions with moisture or through electrochemical reactions during ESI.

6. If you find ions with higher masses than M, look for oligomers of M or other aggregates.

7. Try to assign as many of the intense peaks as possible. However, MS is a highly sensitive technique, and it is easy to get lost in the details of complex spectra. Unless there is a specific ion of interest, assign only analyte peaks that are higher than 10% of the most intense peak. Relative intensities can reflect the composition in solution well, although they are influenced by how easy the molecules ionize, their surface activity, and the ions’ stabilities in the gas phase.

8. The peaks that cannot be assigned with steps 1–5 can be subjected to MS^2^ (see Tandem Mass Spectrometry). Try to track the fragmentation channels of these peaks down at various collision energies and write down the leaving groups, which will add up to the formula of the original ion.

9. If it is not possible to identify any peak related to M, it is likely necessary to change solution conditions and ionization/MS parameters as discussed in the section Technical Details. This could include changing the solvent composition, the charge carrier, the concentration, as well as instrumental parameters for ionization or ion transfer.

## Two-Dimensional Gas Phase Separation Approaches

### Tandem Mass Spectrometry

Most commercial mass spectrometers can perform tandem mass spectrometry (MS^2^) experiments. In an MS^2^ experiment, ion populations are selected based on their *m*/*z* (commonly with a quadrupole) and subsequently activated, which usually results in smaller fragment ions that are in turn mass-analyzed. The activation of ions can be realized via energetic collisions with inert gas (collision-induced dissociation, CID) or surfaces (surface-induced dissociation), electron (electron capture or transfer dissociation) or photon mediation (ultraviolet-photodissociation or infrared multiphoton dissociation), as well as other methods.^80,81^ The most common MS^2^ technique is CID, in which ions are accelerated into a collision cell filled with a stationary inert gas (e.g., N_2_, Ar, Xe). After every gas collision, translational energy of the ion is converted to vibrational energy, which is distributed throughout the ion, usually leading to the dissociation of the weakest bonds. Other tandem mass spectrometry activation methods are not as widespread or not even commercially available and are beyond the scope of this article.^80^

The main information from CID experiments is the composition of fragment ions and indirectly of the leaving groups (fragment mass = precursor mass – leaving group mass), and the stoichiometries of the fragments can inform on the structural subunits present in the original precursor ion and their connectivities. Fragments can further dissociate to secondary fragments, and it is hence important to select collision energies appropriately.

**Figure 3:**
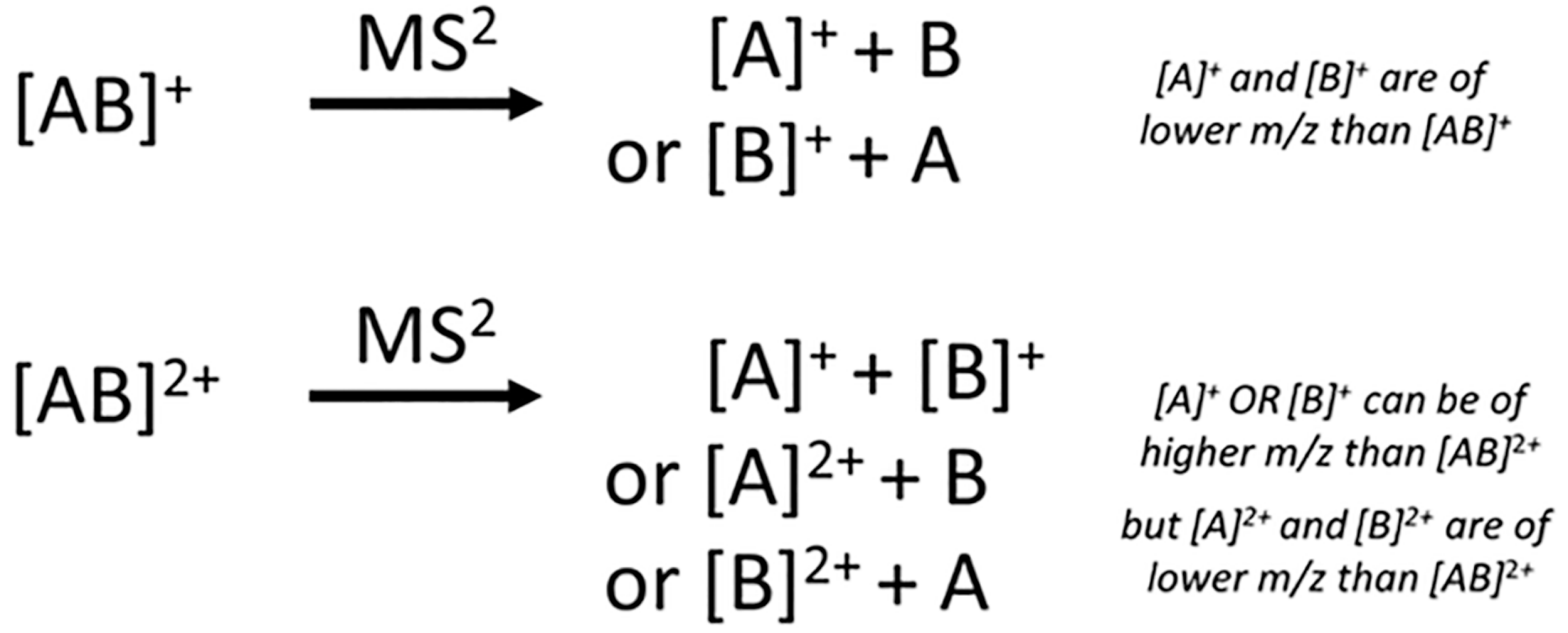
Fragmentation of the singly charged [AB]^+^ and the doubly charged [AB]^2+^. For [AB]^+^, the fragment ions [A]^+^ and [B]^+^ are found at a lower *m*/*z* than the precursor. The dissociation of [AB]^2+^ can result in [A]^+^ or [B]^+^ at a higher *m*/*z* than the precursor, due to the loss of charge, whereas [A]^2+^ and [B]^2+^ are always at a lower *m*/*z* than [AB]^2+^.

The fragmentation of singly charged species occurs from high to low *m*/*z*, involving the loss of neutral leaving groups that lead to singly charged fragment ions. An ion of the structure [AB]^+^ can fragment to [A]^+^ and the neutral B, or to [B]^+^ and A. Both ions [A]^+^ and [B]^+^ are at lower *m*/*z* than the precursor [AB]^+^ (Figure 3). As neutral leaving groups cannot be detected, data visible in the mass spectrum are biased toward structures that retain charge, which is influenced by their size and structure. For the fragmentation of multiply charged species, the charge state *z* can change, leading to fragment ions at higher *m*/*z* (but lower *z*) than the precursor. It is possible that both products of the precursor retain a charge.^19^ An ion [AB]^2+^ can dissociate to [A]^+^ and [B]^+^, and due to the reduction of the charge state *z*, either of these ions (but not both simulatenously) can be at higher *m*/*z* than the precursor (Figure 3).

**Figure 4:**
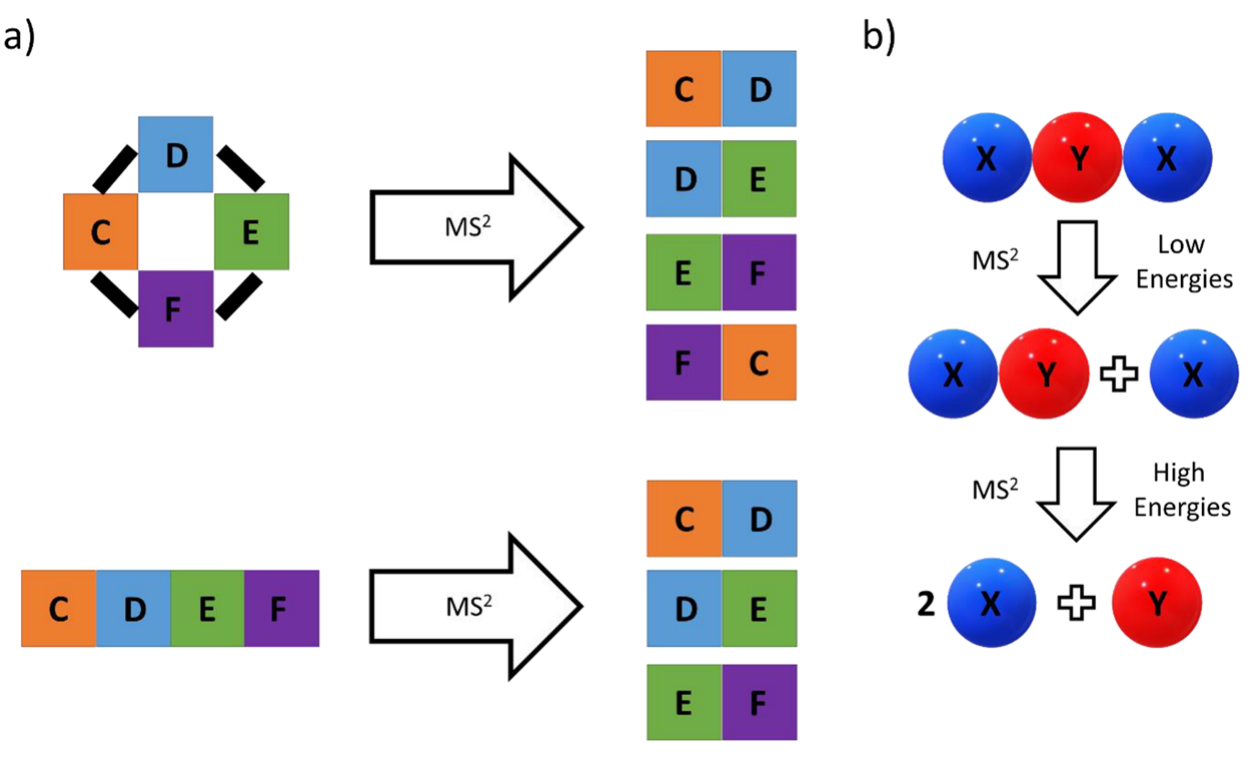
(a) Fragmentation of CDEF with either a cyclic or linear structure. The presence or absence of CF can inform on the connectivity of CDEF. (b) Fragmentation of the chain XYX to the fragments XY + X at lower and 2X + Y at higher collision energies, respectively. The relative abundances of X, Y, and XY can inform on the precursor structure.

In the following paragraphs, two hypothetical cases are discussed in which the analytes consist of the subunits C, D, E, and F and X and Y, respectively (Figure 4). These examples are based on the assumption that all subunits cannot dissociate further, and that the ions are equally likely to retain the charge upon fragmentation.

The precursor ion consists of C, D, E, and F, and the ions CD, DE, EF, and FG are found in the MS^2^ spectrum (Figure 4a, top). From these data, it can be derived that C is likely linked to D and F, D is linked to C and E, E is linked to D and F, and F is linked to E and C. The most sensible explanation is a circular structure of the type CDEF, in which C and F are connected as well, whereas the absence of the ion CF would have indicated a linear connectivity (Figure 4a, bottom). Real examples are more complex, and structural rearrangements can occur and need to be taken into account when deriving structural information from MS^2^ spectra.^82,83^ In practical examples, the units C, D, E, and F might also not be static, and distinguishing the linear from the cyclic species might be possible by measuring differences in the mass of the potentially terminal units C and F.

Another factor is the relative intensities of fragments, which can inform on two points: (1) *Likely fragments statistically occur more often.* In a chain XYX, the fragments X, Y, and XY could be observed (Figure 4b). Fragment Y will likely have the least intense signal at low collision energies, as both X–Y bonds have to be broken. Conversely, both X and XY will occur with similar intensities after the first fragmentation step, as every broken X–Y bond results in equal amounts of X and XY. (2) *Stable fragments are more abundant*. XY is potentially less stable than X as it can dissociate a second time to X and Y, whereas X cannot further fragment. Hence, the relative intensities of the fragments would follow the order X > XY > Y, until at high fragmentation energies XY disappears completely, and twice as many X are present relative to Y (Figure 4b).

The appearance of fragment ions is dependent on the collision voltage, which multiplied by *z* corresponds to the collision energy in the laboratory frame *E*_lab_. This energy is user-defined, and stable ions require higher collision energies to fragment. Varying the energy can investigate ion structure and stability. A good illustrating example is a macrocyle, which can coordinate guests at two different locations: in the inside (“endo”) leading to a host–guest complex, or on the outside (“exo”). Kiesilä et al. investigated the structure of a pyridine[4]arene dimer **Z**_**2**_ that simultaneously coordinates a hexafluorophosphate anion (PF_6_^–^) and an acetone molecule.^84^ Using MS^2^, the authors found that almost exclusively PF_6_^–^ is lost in the first fragmentation step at low collision energies, suggesting that PF_6_^–^ is less strongly bound and hence exo-coordinated (Figure S1, right). In turn, this indicates that acetone is encapsulated into **Z**_**2**_ (“endo”), which was later confirmed by X-ray crystallography (Figure S2). Taken together, this example illustrates how MS^2^ experiments can distinguish different isomers based on their qualitative stabilities.

Stabilities can also be quantified using MS^2^ by ramping the collision energy and plotting it against the “survival yield” (SY), which represents the share of precursor ions that does not fragment. These curves are usually S-shaped, and certain points in the fitted graphs can be determined, e.g. the *E*_50_ value where 50% of the precursor fragments. These values can be regarded as relative measures of ion stability.^85,86^ While they have a thermodynamic meaning for some noncommercial instruments (“guided ion-beam mass spectrometers”),^87^ for commercially available platforms these depend on the instrument, pressures, voltages, and ion structure. The latter is important as large structures experience more collisions and the mass determines the energy that gets transferred during the collisions. These effects can interfere with thermodynamic properties, which is why *E*_50_ and similar values should hence be regarded as semi-quantitative data, which are best used for the interpretation of trends between similar ions.

**Figure 5:**
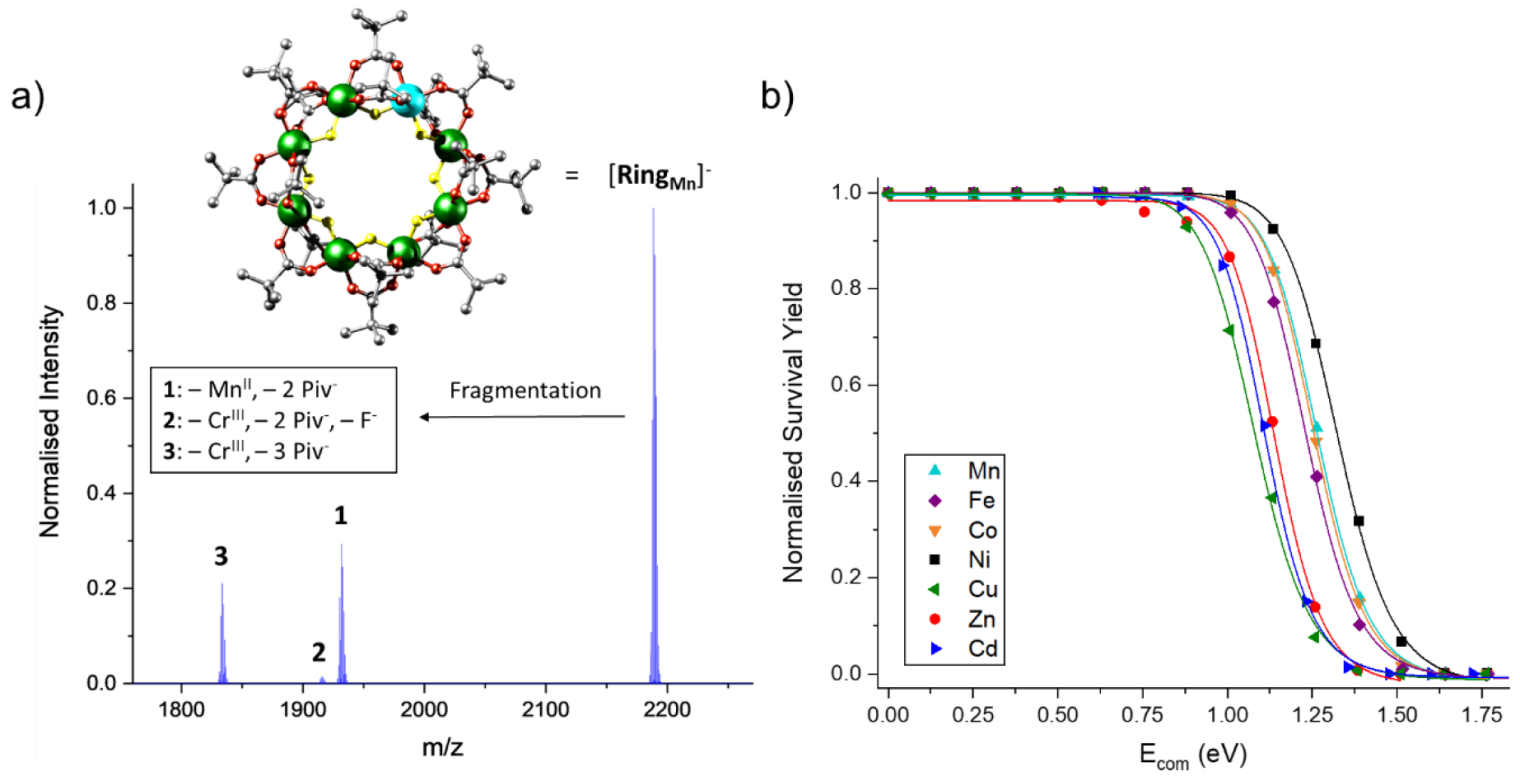
(a) MS^2^ data of [**Ring**_**Mn**_]^−^ at *E*_lab_ = 110 eV. Inset: structure of [**Ring**_**Mn**_]^−^ (Cr, green, Mn, cyan, F, yellow, O, red, C, gray). Hydrogen atoms in the *tert*-butyl groups were omitted for clarity. (b) Normalized survival yield vs *E*_com_ for [**Ring**_**M**_]^−^ fitted to a sigmoidal function (M = Mn, cyan; Fe:, purple; Co, orange; Ni, black; Cu, green; Zn, red; Cd, blue). *E*_com_ is the collision energy in the center-of-mass frame and is a more precise representation of the energy that is transferred during single ion-gas collisions. Reproduced from ref (88). Copyright 2022, The Authors.

One example is the fragmentation and stability trend of the polymetallic rings [Cr_7_M^II^F_8_(O_2_C^*t*^Bu)_16_]^−^ = [**Ring**_**M**_]^−^ (Figure 5a, inset), in which seven Cr^III^ and one divalent metal (M^II^ = Mn^II^, Fe^II^, Co^II^, Ni^II^, Cu^II^, Zn^II^, and Cd^II^) are bridged via fluoride and pivalate ligands (O_2_C^*t*^Bu^–^ = Piv^–^). The dissociation of these anions proceeds through multiple channels, and for [**Ring**_**Mn**_]^−^ this involves the loss of Mn^II^ and two Piv^–^ (to **1**) or the loss of Cr^III^, one F^–^, and two Piv^–^ (to **2**) or Cr^III^ and three Piv^–^ (to **3**; Figure 5a). The isostructural ions with other M^II^’s fragment similarly, and as kinetic effects are likely small, differences in the stability curves (Figure 5b) and *E*_50_ values are likely associated with thermodynamic trends. *E*_50_ differences of up to 22% were found between the most stable M^II^ = Ni^II^ and the least stable Cu^II^, which were rationalized with trends from crystal field theory.^88^

Investigating the stability of compounds is not easily achievable with other techniques, and the unique feature of MS^2^ is that solvent molecules and counterions do not interfere, making it possible to decouple these effects from the actual analyte stability.^6,25^ MS^*n*^ experiments (with *n* > 2) are possible, although not always available in commercial instruments (mostly in instruments with certain types of ion traps). This technique can investigate the disassembly and stability of already fragmented ions.^89^

### Ion Mobility Spectrometry

Ion mobility (IM) is increasingly used in commercial instrumentation, separating ions based on their size, shape, and charge. In the easiest form, ions move through a gas-filled drift cell guided by an electric field. The electric field is usually not strong enough to induce fragmentation; however, collisions with the buffer gas still occur, the more so the larger the ion is. The collisions determine the time the ion spends in the drift cell, and by injecting ion pulses and measuring this time, structural information is gained. The drift (or arrival) time can be converted to a collision cross section (CCS) value, which is comparable across instruments and to values computationally simulated from candidate geometries (e.g., based on density functional theory or molecular dynamics calculations).^9,25^ The community has witnessed a significant increase in the use of IM; however, it is still not available or used in the majority of MS laboratories. In particular, synthetic chemists and facility technicians often do not have access to such platforms, and this technique will hence not be discussed in detail.^9,10,25,90^ The interested reader is referred to a recent perspective on using IM-MS for synthetic molecules.^25^

IM can be an important tool to distinguish isomers, for example binding sites in supramolecules complexes. One illustrating example is the ternary complex with **Z**_**2**_, PF_6_^–^, and acetone that was already discussed above.^84^ MS^2^ showed that the exo-coordinated PF_6_^–^ dissociates more easily from **Z**_**2**_ than acetone, which suggested that PF_6_^–^ is exo- and acetone is endo-coordinated (Figure S1, right). IM can directly give the same information, and measuring the CCS values of several ions involving **Z**_**2**_ showed that those including PF_6_^–^ are larger than those that do not involve PF_6_^–^ (Figure S1, left). As an exo-coordinated species will impact the size of **Z**_**2**_ more than an encapsulated one, this strongly supports that PF_6_^–^ is exo-coordinated, in contrast to acetone.

**Figure 6:**
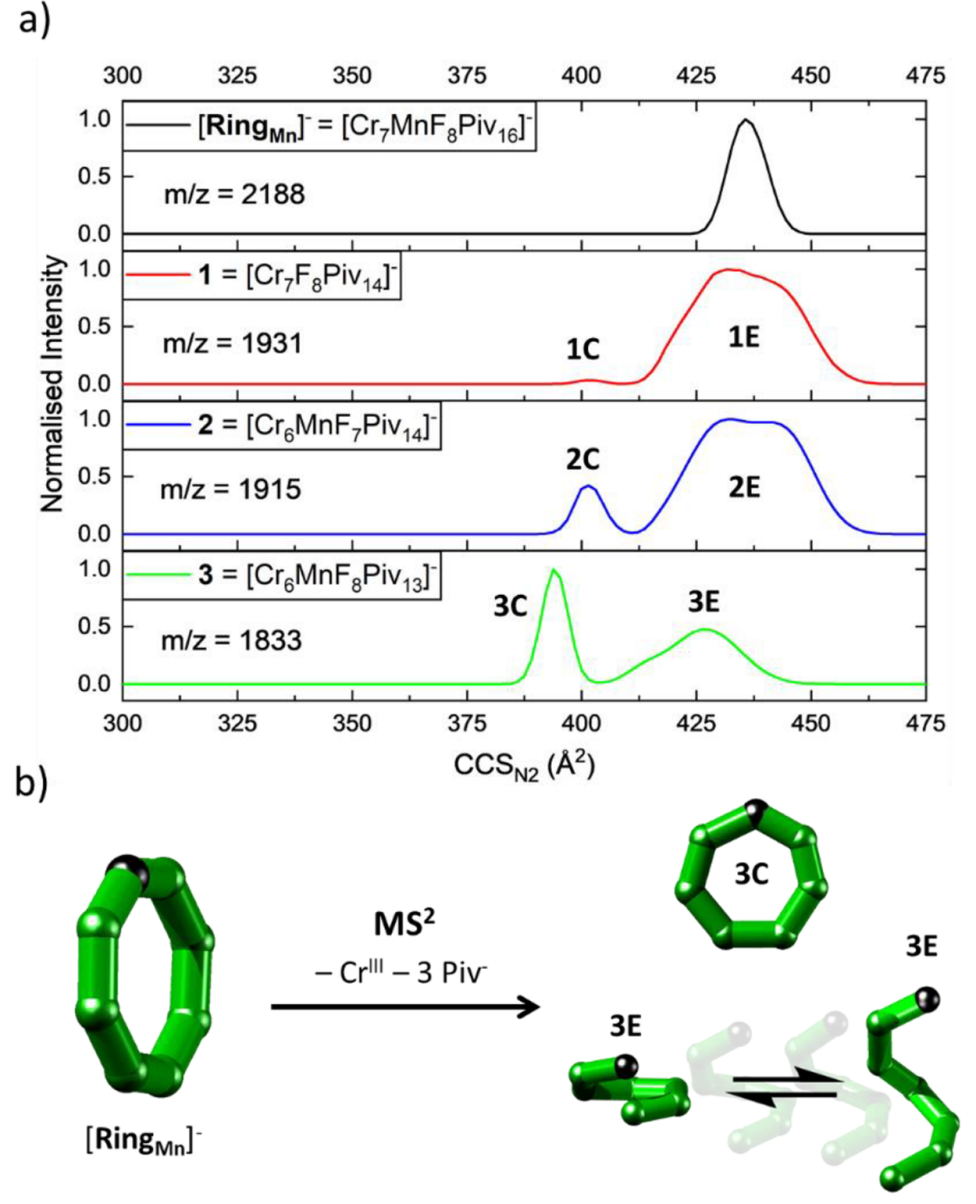
(a) CCS_N2_ Distributions of [**Ring**_**Mn**_]^−^ and fragments (**1**–**3**) at *E*_lab_ = 110 eV. (b) Fragmentation of [**Ring**_**Mn**_]^−^ to **3** including structural assignments of **3C** to closed heptametallic rings and **3E** to conformationally dynamic, open structures. Reproduced from ref (88). Copyright 2022, The Authors.

The study of the polymetallic rings [**Ring**_**M**_]^−^ (Figure 5) further illustrates the insights that IM can offer when combined with MS^2^. The size and shape of fragments **1**–**3** (Figure 6a) were measured, and for all three species, two CCS distributions were found: one at lower CCS and narrow (**C**, “compact”) and one with a wider peak shape at higher CCS (**E**, “extended”). Based on computational structure optimizations and CCS simulations, these were assigned to closed rings (**C**) and conformationally dynamic, open structures (**E**, Figure 6b).^84^ Taken together, these two examples highlight the richness of structural data that can be obtained from IM, and it is anticipated that its application in synthetic laboratories and facilities will significantly increase in the future.^25^

[**Ring**_**M**_]^−^ can encapsulate ammonium cations to form rotaxanes, and the case example of the polymetallic rotaxane **Am**_**Mn**_ will be discussed in the Supporting Information, including sample preparation, MS data analysis, as well as MS^2^ and IM measurements and interpretation.

## Conclusions

The combination of soft ionization sources (mainly ESI), high-resolution mass analyzers, and commercially available MS^2^ and IM additions has enhanced our understanding of coordination compounds and supramolecules. MS is an active field of analytical research, and while the discussion here is limited to techniques that are established and commercially available, other methods such as ion spectroscopy^11,12,91^ or ion soft-landing including microscopic imaging^92,93^ yield further, unique structural information. In my view, all of these techniques will gain in importance for supramolecules and coordination compounds in the future. Overall, this article should be regarded as a guideline on how to make the most of the MS toolbox for labile inorganic compounds, and I hope this tutorial inspires synthetic chemists and technicians to analyze such molecules more frequently and with confidence.

## Supporting Information Available

MS data for the example of the ternary complex involving **Z_2_**; case example for the characterization of the heterometallic rotaxane **Am_Mn_** using different MS methods (PDF)
