# Supplementary material for: Modern Electrospray Ionization Mass Spectrometry Techniques for the Characterization of Supramolecules and Coordination Compounds
Source: Anal Chem. 2024 Apr 30;96(19):7332–41. doi: 10.1021/acs.analchem.4c01028 (PMC11099892; doi:10.1021/acs.analchem.4c01028)
Supplement: Supplementary file 1 — ac4c01028_si_001.pdf [file ac4c01028_si_001.pdf]

Supporting Information

Modern Electrospray Ionization Mass Spectrometry Techniques for the Characterisation of Supramolecules and Coordination Compounds

Niklas Geue*

Michael Barber Centre for Collaborative Mass Spectrometry, Manchester Institute of Biotechnology, Department of Chemistry, The University of Manchester, 131 Princess Street, Manchester, M1 7DN, UK.

Corresponding author: niklas.geue@manchester.ac.uk

Table of Contents

Figure S1: Mass spectrometry data of $\mathbf{Z_2}$ ions. 3

Figure S2: Crystal structure of the ternary complex involving $\mathbf{Z_2}$ 4

Case Example of $\mathbf{Am_{Mn}}$ 5

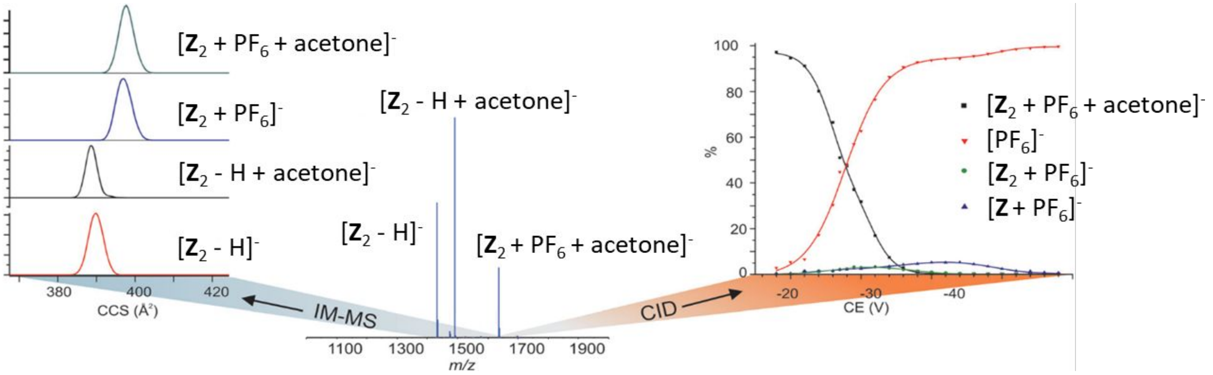

Figure S1: Mass spectrometry data of $\mathbf{Z_2}$ ions. Left: IM-MS data. Those ions that include PF_6^- are significantly larger (higher *CCS*) than those without, suggesting that PF_6^- is *exo*-coordinated in $\mathbf{Z_2}$. Centre: ESI-MS spectrum involving different $\mathbf{Z_2}$ ions. Right: Share of ions vs. collision energy for the precursor $[\mathbf{Z_2} + PF_6 + \text{acetone}]^-$ and fragments, showing the dissociation of PF_6^- in the first fragmentation step at low collision energies. Reproduced from Ref. 1 with permission, © 2017 Wiley-VCH Verlag GmbH & Co. KGaA.

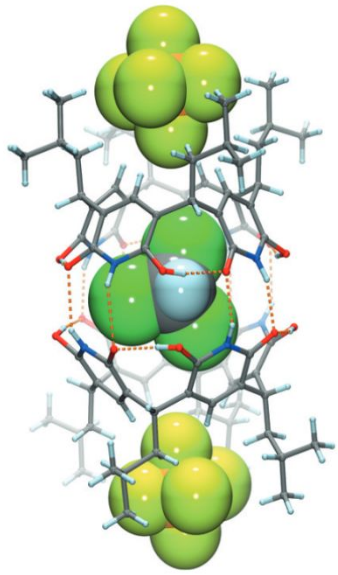

Figure S2: Crystal structure of the ternary complex involving $\mathbf{Z_2}$ with one *endo*-complexed $CHCl_3$ (no figure was available for *endo*-complexed acetone), and two *exo*-coordinated PF_6^-. Hydrogen bonds are shown as dashed orange lines. Reproduced from Ref. 1 with permission, © 2017 Wiley-VCH Verlag GmbH & Co. KGaA.

Case Example of $\mathbf{Am_{Mn}}$

The above discussed [$\mathbf{Ring_M}$]$^-$ are able to encapsulate ammonium cations, and in this detailed case example the data of a polymetallic hybrid inorganic-organic rotaxane with the formula $[NH_2(C_6H_{12}NHC(O)^tBu)_2][Cr_7MnF_8(O_2C^tBu)_{16}]$ = $\mathbf{Am_{Mn}}$ will be discussed, in which the secondary ammonium cation $[NH_2(C_6H_{12}NHC(O)^tBu)_2]^+$ = $\mathbf{TAm^+}$ threads through the polymetallic ring anion $[Cr_7MnF_8(O_2C^tBu)_{16}]^-$ = [$\mathbf{Ring_{Mn}}$]$^-$ (Table S1).[2,3] Previous crystal structure analysis suggested that the *tert*-butyl groups of the cation are too bulky to allow the slipping of the thread through the ring, indicating a kinetically trapped rotaxane structure.[3,4] The monoisotopic mass (sum of the accurate masses of most abundant isotopes) was calculated as 2571.8 Da.

Table S1: Overview of the discussed polymetallic rings [$\mathbf{Ring_M}$]$^-$, the thread $\mathbf{TAm^+}$ and the rotaxanes $\mathbf{Am_M}$. Reproduced from ref. 3, © 2022 The Authors.

	Ring	Thread	Rotaxane
Cartoon of the Discussed Polymetallic Systems	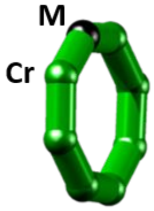	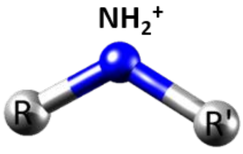	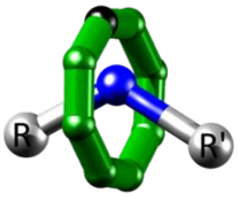
Nomenclature	[$\mathbf{Ring_M}$]$^-$ = $[Cr_7MF_8Piv_{16}]^-$	$\mathbf{TAm^+}$ = $[NH_2RR']^+$	$\mathbf{Am_M}$ = $[NH_2RR'][Cr_7MF_8Piv_{16}]$
Substituents to the Building Blocks	M = Mn^{II}, Fe^{II}, Co^{II}, Ni^{II}, Cu^{II}, Zn^{II}, Cd^{II}	$\mathbf{TAm^+}$: R = R′ = 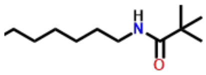	M = Mn^{II}, Fe^{II}, Co^{II}, Ni^{II}, Cu^{II}, Zn^{II}, Cd^{II}<br>$[NH_2RR']$ = $\mathbf{TAm^+}$

The sample of $\mathbf{Am_{Mn}}$ was prepared in 7:3 in a solution of Toluene:MeOH and with 500 µM NaI. The solvent mixture was empirically determined to include a solvent that can dissolve the analyte $\mathbf{Am_{Mn}}$ (toluene) and one that is able to dissolve NaI (methanol), which was used to enhance the signal of the sodiated ion. Sample concentration was set at 10 µM based on standard concentrations used on the instrument of choice (Select Series Cyclic IMS by Waters Corp.).[5]

There are two main regions of interest in the mass spectrum, one between 1240 and 1340 *m/z* and one around 2600 *m/z* (Figure S3a). The most obvious place to look for our analyte with the monoisotopic mass of 2571.8 Da is the latter region, where only +1 ions are present.

As **Am$_{Mn}$** is neutral and NaI was added for signal enhancement, [**Am$_{Mn}$** + Na]$^+$ is the most likely occurring ion. The isotopic pattern was calculated, and showed high agreement with the most intense peak in that region with a maximum at 2595.8 Da (comparable to the agreement in Figure 2a). The second most intense peak is at 2611.8, which is difference of 16 Da. This could correspond to the oxidation with a single oxygen atom, but is also typical for the difference between the alkali metal adducts of Na$^+$ (M = 23 Da) and K$^+$ (M = 39 Da). The former would likely require the presence of a neutral oxygen, as z remains +1, which suggests that the latter is more likely and the ion at 2611.8 Da corresponds to [**Am$_{Mn}$** + K]$^+$. This was confirmed with the simulation of the isotopic distribution and accurate mass, and similarly for the protonated ion [**Am$_{Mn}$** + H]$^+$ at 2573.9 Da.

The most intense peaks are in the region between 1240 and 1340 *m/z*, and these are all doubly charged (Figure S3a Zoom). Their masses are therefore in the region of the molecule **Am$_{Mn}$**, and the assignments [**Am$_{Mn}$** + 2 Na]$^{2+}$ (1309.4 Da), [**Am$_{Mn}$** + Na + K]$^{2+}$ (1317.9 Da) and [**Am$_{Mn}$** + 2 K]$^{2+}$ (1325.9 Da) are hence relatively obvious, confirmed with the simulated isotopic patterns. The ions at 1247.4 Da and 1255.9 Da are also doubly charged, and most likely correspond to the loss of negative charged fragments from **Am$_{Mn}$**. Scientifically, and from mass considerations, the most likely explanation is the loss of one pivalate ligand (M = 101 Da) and the simultaneous addition of an alkali metal cation. Once again, based on comparisons of isotopic patterns, the two ions were identified as [(**Am$_{Mn}$** – Piv) + Na]$^{2+}$ and [(**Am$_{Mn}$** – Piv) + K]$^{2+}$.

The mass spectrum of **Am$_{Mn}$** in positive mode shows no global patterns of repeating units, except for some polymeric units between 750 and 950 *m/z* (Figure S3a). As they are singly charged cations, the distance of 14 *m/z* between the peaks corresponds to 14 *Da*, which is likely due to the subsequent addition of –CH_2– groups. The masses of these species are significantly below the mass of **Am$_{Mn}$**, and are hence not further discussed. Similarly anything in the region below 750 *m/z* only involves singly charged cations, which are most likely contaminations and not relevant for our studies. For example, the most prominent peaks of these regions at 685 and 701 *m/z* were identified as contaminations in plastic ware, as found in the blank mass spectrum. While all the above peaks do not necessarily have to occur, this spectrum highlights how unideal, but real data looks like.

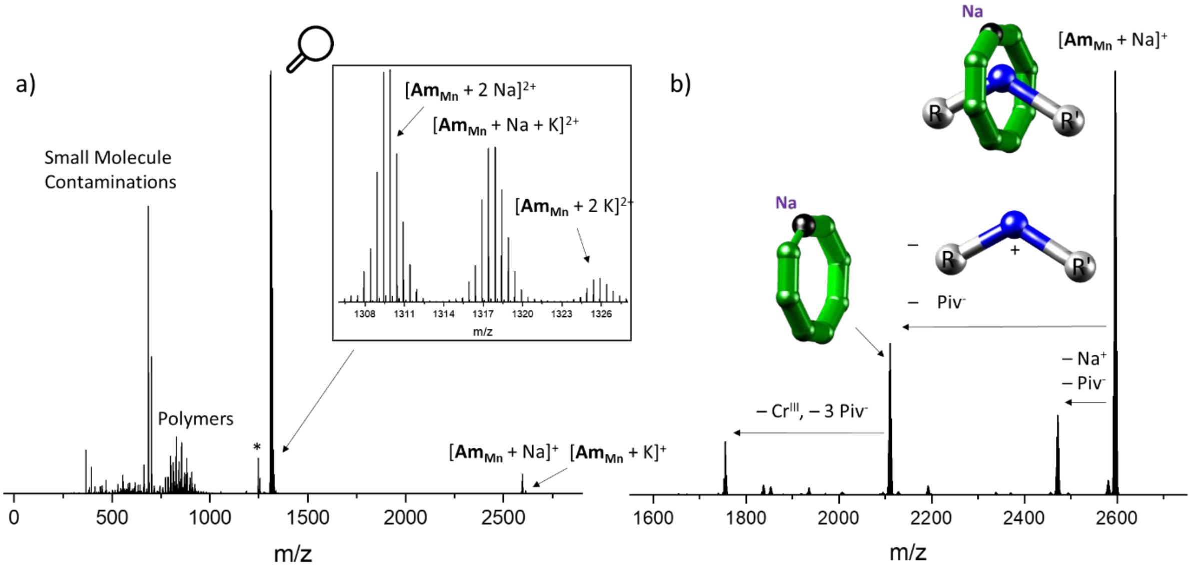

Figure S3: a) Mass spectrum of 10 µM **Am**$_{\mathbf{Mn}}$ in 7:3 Toluene:MeOH with 500 µM NaI including Zoom. Occurring peaks are labelled, including possible small molecule and polymer contaminations. *Ions are assigned as [(**Am**$_{\mathbf{Mn}}$ – Piv) + Na]$^{2+}$ and [(**Am**$_{\mathbf{Mn}}$ – Piv) + K]$^{2+}$. b) MS^2 data from [**Am**$_{\mathbf{Mn}}$ + Na]$^+$ (*m/z* = 2596) at a collision energy of E_{lab} = 100 eV. Fragmentation pathways are labelled, and the dominant primary channel includes the loss of the secondary ammonium thread. Schematic structures of precursor ion and primary fragment are shown (Cr: green, Mn: black, N: blue, C: gray). Reproduced from ref. 3, © 2022 The Authors.

The ions [**Am**$_{\mathbf{Mn}}$ + H]$^+$ and [**Am**$_{\mathbf{Mn}}$ + Na]$^+$ were investigated using CID, and the protonated species fragments showed the loss of a pivalic acid (HPiv) unit, whereas the sodiated ion loses the secondary ammonium cation **TAm**$^+$ and a pivalate ligand as the main channel, leading to [(**Ring**$_{\mathbf{Mn}}$ – Piv) + Na]$^+$ (Figure S3b for sodiated species). The E_{50} values were quantified under comparable instrument conditions, yielding values of E_{50} = 0.25 eV ([**Am**$_{\mathbf{Mn}}$ + H]$^+$) and E_{50} = 1.10 eV ([**Am**$_{\mathbf{Mn}}$ + Na]$^+$), showing a significantly higher stability of the sodiated ion.

IM allowed to understand the structure of both rotaxane ions [**Am**$_{\mathbf{Mn}}$ + A]$^+$ (A = H$^+$, Na$^+$), and their collision cross section distribution revealed that the sodiated species is slightly larger than the protomer.[2] The combination of CID and IM was applied to investigate how a) the structures of the rotaxane ions change upon collisional activation, and b) what structures the fragments exhibit. The sodiated species [**Am**$_{\mathbf{Mn}}$ + Na]$^+$ loses the ammonium cation (Figure S3b), and the mechanism of this disassembly is critical in understanding whether these architectures are pseudorotaxanes, where the ring can slip of the thread, or rotaxanes, where this is not possible and the ring has to break in order to release the thread. IM is a great tool to investigate these questions, however, these discussions are beyond the scope of this article and details can be found in the corresponding publications.[2,3] Overall, this case example

illustrates how the mass spectrum of the polymetallic supramolecule **Am$_{\mathbf{Mn}}$** can be analysed, and how the combination of high-resolution mass spectrometry, tandem mass spectrometry including stability analysis, and ion mobility can characterise its different properties.

References

(1) Kiesilä, A.; Kivijärvi, L.; Beyeh, N. K.; Moilanen, J. O.; Groessl, M.; Rothe, T.; Götz, S.; Topić, F.; Rissanen, K.; Lützen, A.; Kalenius, E. Simultaneous Endo and Exo Complex Formation of Pyridine[4]Arene Dimers with Neutral and Anionic Guests. *Angew. Chem. Int. Ed.* **2017**, *56* (36), 10942–10946. https://doi.org/10.1002/anie.201704054.

(2) Geue, N.; Bennett, T. S.; Ramakers, L. A. I.; Timco, G. A.; McInnes, E. J. L.; Burton, N. A.; Armentrout, P. B.; Winpenny, R. E. P.; Barran, P. E. Adduct Ions as Diagnostic Probes of Metallosupramolecular Complexes Using Ion Mobility Mass Spectrometry. *Inorg. Chem.* **2023**, *62* (6), 2672–2679. https://doi.org/10.1021/acs.inorgchem.2c03698.

(3) Geue, N.; Bennett, T. S.; Arama, A. A.; Ramakers, L. A. I.; Whitehead, G. F. S.; Timco, G. A.; Armentrout, P. B.; Mcinnes, E. J. L.; Burton, N. A.; Winpenny, R. E. P.; Barran, P. E. Disassembly Mechanisms and Energetics of Polymetallic Rings and Rotaxanes. *J. Am. Chem. Soc.* **2022**, *144* (49), 22528–22539. https://doi.org/10.1021/jacs.2c07522.

(4) Ballesteros, B.; Faust, T. B.; Lee, C. F.; Leigh, D. A.; Muryn, C. A.; Pritchard, R. G.; Schultz, D.; Teat, S. J.; Timco, G. A.; Winpenny, R. E. P. Synthesis, Structure, and Dynamic Properties of Hybrid Organic-Inorganic Rotaxanes. *J. Am. Chem. Soc.* **2010**, *132* (43), 15435–15444. https://doi.org/10.1021/ja1074773.

(5) Giles, K.; Ujma, J.; Wildgoose, J.; Pringle, S.; Richardson, K.; Langridge, D.; Green, M. A Cyclic Ion Mobility-Mass Spectrometry System. *Anal. Chem.* **2019**, *91* (13), 8564–8573. https://doi.org/10.1021/acs.analchem.9b01838.
